# Copper and bezafibrate cooperate to rescue cytochrome *c* oxidase deficiency in cells of patients with *sco2* mutations

**DOI:** 10.1186/1750-1172-7-21

**Published:** 2012-04-19

**Authors:** Alberto Casarin, Gianpietro Giorgi, Vanessa Pertegato, Roberta Siviero, Cristina Cerqua, Mara Doimo, Giuseppe Basso, Sabrina Sacconi, Matteo Cassina, Rosario Rizzuto, Sonja Brosel, Mercy M Davidson, Salvatore DiMauro, Eric A Schon, Maurizio Clementi, Eva Trevisson, Leonardo Salviati

**Affiliations:** 1 Clinical Genetics Unit, Dept. of Pediatrics, University of Padova, Via Giustiniani 3, Padova, 35128, Italy; 2 Dept. of Biomedical Sciences, University of Padova, Padova, Italy; 3 Hematology-Oncology Laboratory, Dept. of Pediatrics, University of Padova, Padova, Italy; 4 Centre de référence des Maladies neuromusculaires and CNRS UMR6543, Nice University Hospital, Nice, France; 5 Departments of Neurology, Columbia University, New York, NY, USA; 6 Department of Genetics and Development, Columbia University, New York, NY, USA

**Keywords:** COX deficiency, Bezafibrate, *SCO2*, Copper chaperones, Copper supplementation

## Abstract

**Background:**

Mutations in *SCO2* cause cytochrome *c* oxidase deficiency (COX) and a fatal infantile cardioencephalomyopathy. *SCO2* encodes a protein involved in COX copper metabolism; supplementation with copper salts rescues the defect in patients’ cells. Bezafibrate (BZF), an approved hypolipidemic agent, ameliorates the COX deficiency in mice with mutations in *COX10*, another COX-assembly gene.

**Methods:**

We have investigated the effect of BZF and copper in cells with *SCO2* mutations using spectrophotometric methods to analyse respiratory chain activities and a luciferase assay to measure ATP production.*.*

**Results:**

Individual mitochondrial enzymes displayed different responses to BZF. COX activity increased by about 40% above basal levels (both in controls and patients), with *SCO2* cells reaching 75-80% COX activity compared to untreated controls. The increase in COX was paralleled by an increase in ATP production. The effect was dose-dependent: it was negligible with 100 μM BZF, and peaked at 400 μM BZF. Higher BZF concentrations were associated with a relative decline of COX activity, indicating that the therapeutic range of this drug is very narrow. Combined treatment with 100 μM CuCl_2_ and 200 μM BZF (which are only marginally effective when administered individually) achieved complete rescue of COX activity in *SCO2* cells.

**Conclusions:**

These data are crucial to design therapeutic trials for this otherwise fatal disorder. The additive effect of copper and BZF will allow to employ lower doses of each drug and to reduce their potential toxic effects. The exact mechanism of action of BZF remains to be determined.

## Background

Cytochrome *c* oxidase (COX), complex IV of the mitochondrial respiratory chain (RC), is comprised of 13 structural subunits and a number of prosthetic groups and metal cofactors [1]. For its biogenesis, COX requires several ancillary proteins encoded by COX assembly genes, which are needed for the synthesis of the prosthetic groups, for the delivery of the metal cofactors, and for the stabilization of the nascent polypeptides [2]. Mutations in COX assembly genes (mainly *SURF1, SCO2,* and *COX10*) are the most common cause of isolated COX deficiency [3]. These disorders are characterized by severe encephalomyopathy, and involvement of other tissues (hypertrophic cardiomyopathy in *SCO2* patients [4], tubulopathy and/or cardiomyopathy in *COX10* patients [5]), and usually leads to death in infancy or early childhood. There is no established therapy for these conditions.

*COX10* is required for heme biosynthesis [6], while the precise function of *SCO2* and *SURF1* is still debated. Homologues of *SURF1* in bacteria appear to function as heme-binding proteins [7], while SCO2 is implicated in copper metabolism although its function is not completely clear [4,8]. SCO2 itself is a copper-binding protein, and it has been shown that copper supplementation can rescue COX deficiency in cells harboring mutations in this gene [9,10]. Although a preliminary trial in patients yielded promising results [11], and supplementation with copper salts is currently employed in patients with Menkes disease [12], the potential toxicity of these compounds is a major hindrance to their therapeutic use.

A novel approach for the treatment of RC disorders is based on induction of mitochondrial biogenesis. This was achieved in experimental models by overexpression of PPARgamma -coactivator alpha (PGC-1alpha), or by pharmacological treatment. Bezafibrate (BZF), an approved hypolipidemic agent, was originally employed in patients with disorders of mitochondrial fatty acid metabolism with promising results [13]. It was later shown to be effective also in RC disorders. Mice with mitochondrial myopathy due to muscle-specific knockout of the *COX10* gene displayed increased muscle COX activity and ATP levels, delayed onset of myopathy, and markedly prolonged life span [14]. BZF proved effective also in cells of patients with COX deficiency [15]. Another approach is based on 5-aminoimidazole-4-carboxamide ribonucleoside (AICAR). This compound acts by activating AMP-activated protein kinase [16], which in turn stimulates PGC-1alpha expression. AICAR was found to be effective in mice with mutations in *SURF1SCO2*, and *COX15 *[17], whereas BZF was not effective in mice and was associated with significant toxicity in both control and COX-deficient animals. However, the response to BZF is a species specific phenomenon, and hepatomegaly after BZF treatment is typical of rodents but is not usually seen in other mammalian species, including humans [18,19]. Moreover, AICAR is still not approved for human use, while BZF is routinely employed in clinical practice. We therefore examined the effect of BZF on a cellular model of *SCO2* deficiency to see if this drug could have a role for the treatment of patients with *SCO2* mutations.

## Materials and methods

### Reagents and cell lines

Cell culture reagents were purchased from Invitrogen. All other chemicals were purchased from Sigma.

Cell lines used in this work have been described previously [10]. Briefly, Pt 1 and Pt 2 were compound heterozygotes for the common *SCO2* mutation E140K and for a truncating mutation. Pt 3 was a compound heterozygote for two truncating *SURF1* mutations. Patients with coenzyme Q_10_ deficiency have been described previously [20].

### Cell culture

Cell lines were cultured in Dulbecco modified Eagle medium (DMEM) (Invitrogen) supplemented either with 20% fetal bovine serum (FBS) (primary skin fibroblasts) or with 10% FBS (all other cell lines), 100 mg/mL streptomycin, and 100 units/mL penicillin.

### Copper and BZF supplementation

BZF was solubilised in DMSO to obtain a 100 mM stock solution. This stock solution was further diluted with DMSO to the desired concentration. The total dose of DMSO solvent in the culture medium was 0.5% for all samples (i.e. 60 μL in 12 mL of medium). CuCl_2_ was mixed in the medium before the addition of serum in order to avoid precipitation. Cells were treated with BZF alone for five days, whereas BZF + CuCl_2_ supplementation experiments were carried out for 10 days [10].

### Biochemical assays

RC complexes were analyzed using standard spectrophotometric assays as described [21,22]. Results were normalized to total protein, and to citrate synthase (CS). However, in copper supplementation experiments, activities were normalized only to protein, because CS activity is inhibited by high copper concentrations [10]. Ornithine aminotransferase (OAT) activity was measured as described [23]. Statistical significance was calculated as previously reported [10].

### Immunoblot analysis

Equal amounts of total cell proteins were separated by PAGE using 4-12% gradient gels and tris-glycine buffer (Invitrogen). After blotting, membranes were probed either with an antibody against COXII (Molecular Probes) or with an antibody against SCO2 (Santa Cruz), and with an anti actin antibody (Sigma) according to the manufacturer’s protocol. Detection was performed using the ECL advanced kit (GE biosciences). Densitometry was performed using the ImageJ software.

### Luminescence measurements

Luciferase assay was carried out, as previously described [24]. In brief, wild type or *SCO2* mutant fibroblasts (200,000-300,000 per coverslip) were grown in the presence or absence of 400 μM BZF for four days. They were then transfected with mitochondrial targeted luciferase (mtLUC) using the Amaxa Nucleofector apparatus (Lonza) and a standard electroporation procedure. The cells were then treated for additional 24 h. The coverslips were transferred to the 37°C thermostated chamber of a luminometer and perfused with a Krebs Ringer Buffer containing: 125 mM NaCl, 5 mM KCl, 1 mM Na_3_PO_4_, 1 mM MgSO_4_, 20 μM Luciferin, 20 mM Hepes, 5,5 mM Glucose (pH7,4). Luminescence is entirely dependent on the continuously provided luciferin and proportional to ATP concentration (between 20 and 200 μM). After 60 s equilibration in the new medium, during which the light emission of mitochondrial luciferase-transfected cells was in the range of 500–5000 cps versus a background lower than 10 cps, cellular response was evoked by adding the agonist histamine (100 μM) to the perfusion medium.

### Cell death and ROS measurements

4.5x10^4^ cells grown in 12-well plates were treated with BZF at the indicated concentration and after 4 days treated with 1 mM H_2_O_2_ for 2 h . Cells were stained with propidium iodide (PI) and annexin-V-FITC (Prodotti Gianni) and cell death was measured by flow cytometry as the percentage annexin-V, PI-positive cells [25]. ROS production was assayed using dichlorodihydrofluorescein diacetate and flow cytometry as described [26].

## Results

### BZF stimulates the activity of individual RC complexes

**Figure 1:**
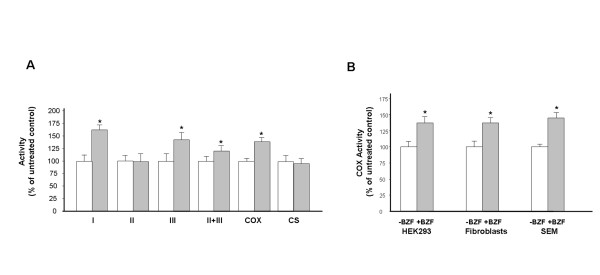
**A) RC enzyme activities in HeLa Cells treated with 400 μM BZF.** HeLa cells were incubated for 5 days with 400 μM BZF and then assayed for RC complex activities. * = significant difference versus untreated (*p*<0.05). **B)** HEK293, primary skin fibrolasts and SEM cells (a leukaemia cell line) were treated with 400 μM BZF as in panel A and then assayed for COX activity. We did not detect significant changes in CS activity in any cell line after BZF tretment.

To study the effect of BZF treatment on the activity of individual RC complexes, we incubated HeLa cells with the reportedly effective dose of 400 μM BZF [14] for five days. Cells were then harvested and RC enzyme activities were measured on lysates. Activities of individual enzymes were stimulated differently: the highest increase was in the activity of complex I, while complex II was unaffected, and the effect on complex III and IV activity was intermediate (Figure 1A). We did not detect significant variation also in the activities of citrate synthase (CS) and of another mitochondrial matrix enzyme unrelated to energetic metabolism, OAT (not shown).

### BZF induces comparable increase of COX activity in different cell lines

Next, we focused on the effect on COX in different cell lines: HEK293 cells, primary skin fibroblasts from a healthy individual, and bone marrow-derived SEM cells. We detected similar increases of COX activity (+37%, +38%, and +43%) in the three cell types compared to untreated samples (Figure 1B). In all cases, COX activity was virtually 100% KCN-sensitive. Again we did not detect significant variations of CS activity.

### BZF stimulates COX activity in SCO2 cells in a dose-dependent but peaks at 400 μM

We then analysed the effect of BZF in normal and COX-deficient fibroblasts. Although this cell model is not ideal because fibroblasts display only partial COX deficiency (COX activity around 50% of controls), *MyoD*-transformed fibroblasts, myoblasts or myotubes obtained from these patients also displayed only partial COX deficiency [27] and we detected the full COX-deficient phenotype only in fully differentiated muscle. A similar phenomenon was noted with other assembly factors such as *FOXRED1 *[28]*.* Therefore these cell types do not provide significant advantages compared to skin fibroblasts for our studies.

**Figure 2:**
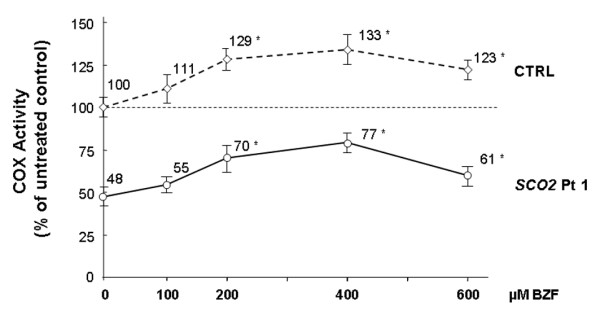
**Primary skin fibroblasts of a normal control and of a patients with*SCO2*mutations were incubated with 0, 100, 200, 400, and 600 μM BZF for 5 days and then assayed for COX activity.** * = significant difference versus untreated (*p*<0.05). ** = significant versus 400 μM. Data are expressed normalized to protein, but there was no variation when they were normalized to CS activity.

**Figure 3:**
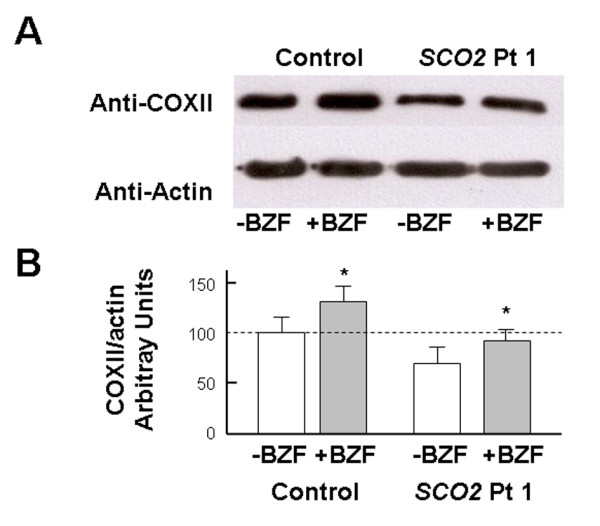
**A) Lysates of cells treated with 400 μM BZF were separated on a 4-12% gradient gel in a SDS-tris-glycine buffer.** After blotting, membranes were probed with anti COXII and anti-actin antibodies. **B)** densitometric analysis of the previous gel (three different experiments). Results are normalized to beta actin levels.

To explore the dose-dependency of the treatment, we incubated *SCO2* mutant and control cells with increasing doses of BZF (0, 100, 200, 400, 600 μM BZF) for five days. With 100 μM BZF only a minimal response was noted in both patient and controls (Figure 2), while there was an increase of COX activity in both patient and control, with a peak at 400 μM BZF, and a relative decline at 600 μM BZF in both cell types (Figure 2). We did not detect significant changes in CS activity even in this case. In both cell types, the increase in COX activity was paralleled by a similar increase in the steady state levels of Cox2p (Figure 3A and 3B).

**Figure 4:**
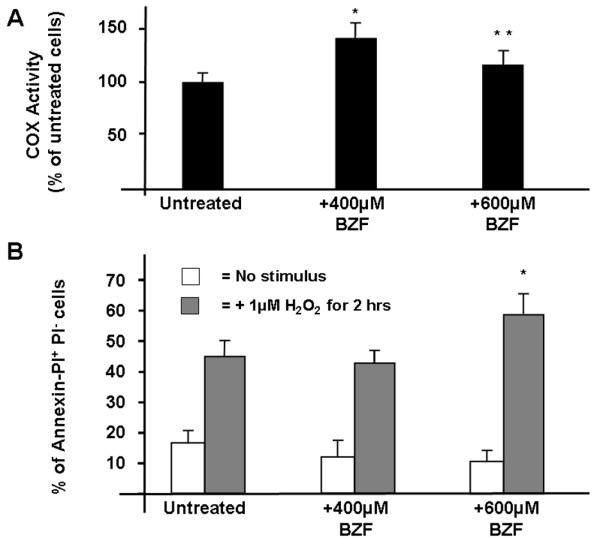
**A) COX activity in HeLa cells treated with increasing doses of BZF**. **B)** Percentage of apoptotic cells under basal conditions or after incubation with 1 mM H_2_O_2_ for 2 h.

We considered whether the decline at higher BZF concentrations could be caused by increased production of reactive oxygen species (ROS), but we did not detect significant variations of ROS in BZF treated cells (not shown). This type of response to BZF was noted also in HeLa cells (Figure 4A). In this case we analyzed also cell death. Under basal conditions there was no increase of apoptotic cells after BZF treatment, and we detected a minor (albeit significant) increase in annexin-positive cells after treatment with 600 μM BZF only after stimulation with H_2_O_2_ (Figure 4B).

**Figure 5:**
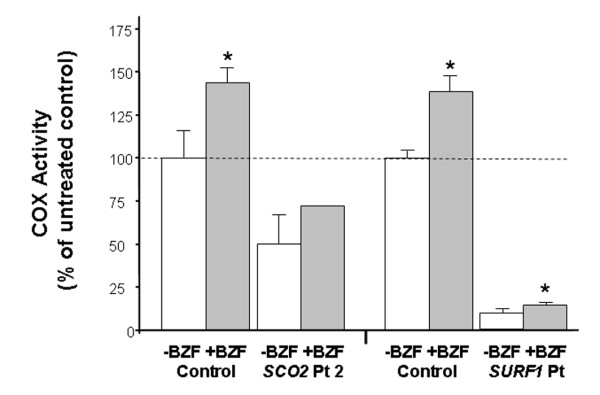
**COX activity in fibroblasts of a second*SCO2*patient and of a*SURF1*patient were treated with 400 μM BZF for five days.** In *SCO2* patient 2 the analysis could be performed only in duplicate in BZF-treated cells. * = significant versus untreated (*p*<0.05).

Because of the effect on COX activity observed in these experiments, we treated with 400 μM BZF fibroblasts from two other patients, one with *SCO2* mutations and one with *SURF1* mutations. We confirmed the effect on the second *SCO2* patient (+38%), whereas in the *SURF1* cells, although the relative increment of COX activity was similar (+40%), the absolute increase was much smaller (from 10% to 14% residual activity compared to untreated controls) (Figure 5).

We confirmed the lack of stimulation of complex II in cultured skin fibroblasts and we did not detect any effect on CoQ_10_ content in CoQ10 deficient fibroblasts (not shown).

### BZF increases ATP production in fibroblasts with SCO2 mutations

A mitochondrially targeted chimera of ATP-sensitive photoprotein Luciferase (mtLUC) was exploited to dynamically monitor ATP synthesis within the mitochondrial subcellular compartment. The method is based on the reaction of luciferase with the substrate luciferin in presence of oxygen, as the resulting light emission is a linear function of ATP concentration in a range between 10^-3^ and 10^-2^ M. *SCO2* fibroblasts and normal controls were grown for 5 days in the presence of 400 μM BZF (see methods). ATP production was triggered by addition of 100 μM histamine to the medium.

**Figure 6:**
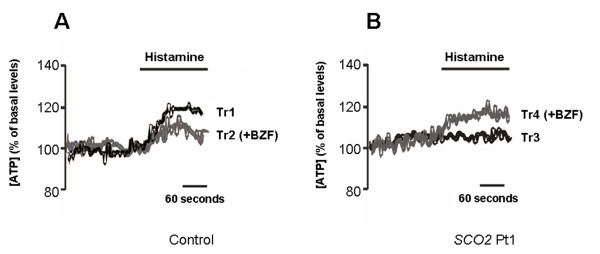
**Measurements of mitochondrial ATP concentration in control fibroblasts (A) and*SCO2*cells (B).** Where indicated the cells were challenged with 100 μM histamine, black and grey traces refer to BZF treated and untreated cells. The panel shows representative traces of 20 coverslips for each condition in 3 different experiments. Trace 1 (Tr1) = untreated control; Tr2 = control + 400 μM BZF; Tr3 = untreated *SCO2* patient; Tr4 = *SCO2* patient + 400 μM BZF.

The increases [Ca^2+^ after stimulation with histamine in the mitochondrial matrix cause an increase in ATP levels due to the stimulation of Ca^2+^-dependent dehydrogenases. This increment is impaired in cells with intrinsic mitochondrial defects, such as the mitochondrial DNA 8344 A > G point mutation in the tRNA^Lys^ gene [29]. In accordance with these data we observed an increase of ATP levels in mitochondria of normal cells (+19.1 ± 3% compared to basal levels) after stimulation with histamine (Figure 6A, Tr1), while this increment was absent in *SCO2* cells (Figure 6B, Tr3).

We then evaluated the effect of BZF treatment. In control cells, there was a reduction of the ATP increase after stimulation with histamine (+10.5± 2% of the basal levels) compared to untreated cells, (Figure 6A, Tr2), similar to what has been observed in cells overexpressing PGC*-*1alpha, which display a reduction of Ca^2+^ transients in the mitochondrial matrix [30] with reduced stimulation of matrix dehydrogenases and a less evident rise in ATP levels upon stimulation. In *SCO2* mutant cells, we observed the opposite effect, as BZF treatment caused a significant increase in ATP levels (+10.4 ± 2% of basal levels) after stimulation with histamine (Figure 6B, Tr3). These data suggest that the biochemical defect was significantly restored, with a detectable effect on the functional output of mitochondria.

### BZF and CuCl_2_ display an additive effect in rescuing COX deficiency in SCO2 cells

**Figure 7:**
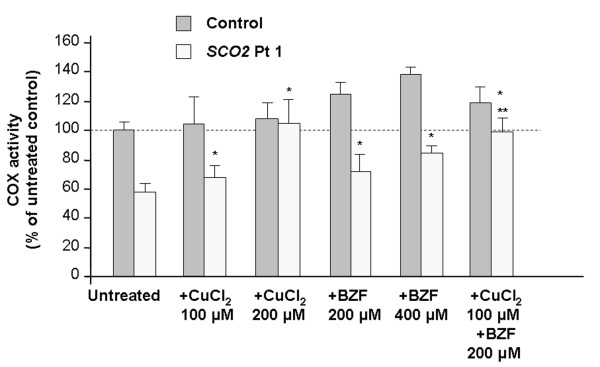
**Additive effect of copper and BZF.** Cells were treated with 200 μM BZF, 400 μM BZF, 100 μM CuCl_2_, 200 μM CuCl_2_, or with 200 μM BZF plus 100 μM CuCl_2_ for 10 days. * = significant versus untreated (*p*<0.05). ** = significant versus untreated and versus cells treated with 100 μM CuCl_2_ or 200 μM BZF (*p*<0.05).

Copper was previously shown to rescue COX deficiency in *SCO2* mutant cells in a dose-dependent manner with a maximum effect at 200 μM CuCl_2_, and the effect required more than a week to become evident [10]. We compared the effect of BZF and CuCl_2_, individually and in combination. As seen in Figure 7, incubation with suboptimal doses of either compound (100 μM CuCl_2_ or 200 μM BZF) caused only a minor increase of COX activity, while incubation with the two compounds together resulted in complete recovery of COX activity in patient cells, similar to the effect of 200 μM CuCl_2_ alone (and much more evident than the effect of 400 μM BZF alone). Moreover, longer incubation (10 versus 5 days) with BZF alone did not result in higher COX activity compared to what we observed in previous experiments.

### Effect of CuCl_2_ or BZF treatment on SCO2 protein levels

**Figure 8:**
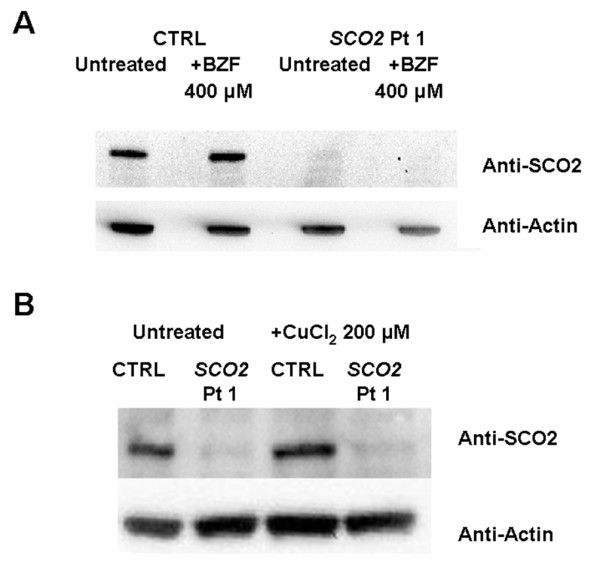
**Western blot analysis of SCO2 in cells treated with 400 μM BZF (A) or 200 μM CuCl_2_(B) for 10 days.** Beta actin was used as loading control.

Western blot analysis showed a marked reduction of SCO2 protein consistent with the notion that the E140K mutant is unstable [9,31] (Figure 8A and 8B). Treatment with BZF did not significantly alter SCO2 levels in both patient or control cells (Figure 8A), indicating that the increase in COX activity observed is not mediated by induction of *SCO2* expression. We did not detect a marked variation of SCO2 also after CuCl_2_ treatment (Figure 8B). However densitometric analysis of three different experiments did detect a small (but statistically significant , p < 0.05) increase in the levels of SCO2 from 9 ± 5% of controls to 16 ± 7% of controls after treatment with 200 μM CuCl_2_. These data must be taken with caution because the SCO2 band in the patient is very faint and densitometric analysis may be prone to errors, nevertheless these finding could indicate that copper supplementation may indeed function at least in part through stabilization of the mutant protein. The modest increase in SCO2 could explain the long incubation time (about 1 week) required to achieve complete correction of COX deficiency in these cells [27].

## Discussion

With the exception of coenzyme Q_10_ deficiency [32], there is currently no established treatment for patients with RC defects. Pharmacological stimulation of the biogenesis of the RC is a promising new approach for the treatment of these disorders. Two compounds have been shown to be effective to this purpose. BZF, which has been successfully employed in patients with defects of mitochondrial beta-oxidation, was shown to be effective also in a mouse model of COX deficiency due to *COX10* mutations [14], and in cells of patients with COX deficiency due to *COX10* mutation and with unknown defects. However a recent study could not confirm the efficacy of BZF in mice with defects in *SCO2COX15*, or *SURF1*, and it was found to cause severe hepatomegaly in both control and COX-deficient animals, whereas AICAR, another activator of the PPARgamma/PGC-1alpha pathway, could rescue the phenotype in these animals [17].

Yet, these negative results concerning the efficacy of BZF should be taken with caution for two reasons. Mice (and rodents in general) are not a good model to study BZF. In fact it has been shown that there are marked species-specific differences in the effects of BZF, which is known to cause hepatomegaly in mice and rats, but not in other mammals such as dogs and monkeys [18] (or humans). The biological bases of this phenomenon are not clear. In fact, BZF is commonly used in patients and elevation of liver enzymes is an infrequent side effect in clinical use [19]. Moreover, our results show that the therapeutic window for BZF is very narrow (see below), therefore dosage is a critical issue when administering BZF to animals (or patients), and excessive doses may result in lack of response. A possible explanation for the observed lack of effectiveness of BZF in the COX deficient mice models, could be that the plasma levels of the drug fell outside the narrow therapeutic range (animals were administered BZF in drinking water and serum levels were not monitored). As pointed out by Djouadi and Bastin mice received a dose of BZF 100-fold the therapeutic dose used in patients [33].

Although the effects of AICAR in COX deficient mice were striking, this compound is still not approved for medical use, while BZF and copper are routinely used in patients. We have therefore examined the effectiveness of BZF (with or without copper) in human cells with *SCO2* deficiency, to assess the potential use of these drugs in patients with *SCO2* mutations. Our data support the efficacy of the combined treatment with these two compounds. Although in principle we cannot rule out the hypothesis that the effect we observed occurs only in cultured cells, and not in whole tissues, nevertheless patients with beta oxidation defects receiving BZF therapy displayed an increase in COX activity in their muscles [33]. Our results highlight some important points that should be taken into account when designing experiments in non rodent animal models, or therapeutic trials in patients.

First, the response of COX activity to BZF treatment was similar (an increase of about 40% compared to baseline levels) in all cell lines we studied (primary fibroblasts, HeLa, HEK293, and SEM cells), and the same relative increase was also detected in COX-deficient cells (both in *SCO2* and *SURF1* mutants), indicating that BZF does not increase efficiency of the assembly process, but simply stimulates COX biogenesis as a whole. The increase of COX activity after BZF treatment was accompanied by increase of cellular ATP production in *SCO2* cells.

However, the precise mechanism of action of BZF is unclear [34]. In fact we detected an increase in enzymatic activities of complex I, III, and IV, whereas other mitochondrial proteins, both matrix enzymes such as CS and OAT, or localized to the mitochondrial inner membrane, such as complex II and SCO2 protein itself, were unaffected by BZF treatment, ruling out an effect of BZF on mitochondrial biogenesis as a whole. Coenzyme Q_10_ levels in deficient cells were similarly unaffected. BZF appears to be stimulating only RC complexes which contain mitochondrial DNA encoded subunits, and not mitochondrial biogenesis as a whole. These findings argue against an involvement of the PGC1alpha pathway, but the exact mechanism of action remains to be elucidated. The apparently different mechanism of action of AICAR and BZF suggest that these two compound could also be used in synergy. Future work will be aimed to test this hypothesis.

Second, we noted that the effect of BZF on COX activity peaks at a BZF concentration of 400 μM in the culture medium. However, higher BZF concentrations (600 μM) do not result in a plateau of the effect, but we observed instead a relative reduction of COX activity. A slight decrease of COX activity with 500 μM BZF was in fact noted also by Bastin and coworkers [15] and they did not test higher BZF doses. These results are critical for therapeutic trials, because they imply that plasma BZF levels must be closely monitored in patients, since the therapeutic window for this compound appears to be very narrow, and doses too high or too low will be ineffective. The reason for the observed decrease in efficacy at higher BZF concentrations is not clear. We observed a minor increase in susceptibility to apoptosis in cells treated with 600 μM BZF, but only after incubation with H_2_O_2_. Some sort of negative feedback mechanism could be acting, but further work is needed to address this issue.

Third, and most important, we demonstrated a synergistic effect between copper and BZF. The effect of BZF alone is relatively modest, but we had shown previously that CuCl_2_ can rescue COX activity in *SCO2* mutant cells in a dose-dependent manner with complete recovery of COX activity at 200 μM CuCl_2 _[10]. Preliminary trials in patients have also yielded promising results, although one major drawback is the toxicity of copper [11]. Our present data demonstrate that CuCl_2_ and BZF have an additive effect, which permits to employ lower doses of each compound and still achieve complete normalization of COX activity in patient’s cells. It will be possible to avoid at least part of the toxicity related to high serum copper levels, while employing BZF at doses lower than the peak effective levels of 400 μM.

## Conclusions

Taken together, our data support the use of BZF in combination with copper in patients with *SCO2* mutations. We underscore the fact that both compounds are routinely used in patients with other diseases therefore their utilization for *SCO2* patients would simply represent an off-label use. We also stress the importance for clinicians, in particular pediatric neurologists and cardiologists, to consider the possibility of *SCO2* mutations in newborns and infants presenting with hypertrophic cardiomyopathy of unexplained origin. Early diagnosis is essential for the ultimate outcome of patients because, as we learned from children with CoQ_10_ deficiency [35], effective treatments may stop the progression of the neurological disease but will not affect the already established cerebral lesions.

## Abbreviations

AICAR = 5-aminoimidazole-4-carboxamide ribonucleoside; BZF = bezafibrate; COX = cytochrome c oxidase; CS = citrate synthase; OAT = ornithine aminotransferase; PGC-1alpha = PPARgamma -coactivator alpha; PPAR = peroxisome proliferator-activated receptor; RC = respiratory chain.

## Competing interests

The authors declare that they have no competing interests

## Authors’ contributions

Alberto Casarin Performed experiments, Gianpietro Giorgi Performed experiments, Vanessa Pertegato Performed experiments, Roberta Siviero Performed experiments, Mara Doimo Performed experiments, analysed data, Cristina Cerqua Performed experiments, Giuseppe Basso Analysed data, revised manuscript, Sabrina Sacconi Designed experiments, analysed data, Matteo Cassina Analysed data, revised manuscript, Rosario Rizzuto Designed experiments, analysed data, revised manuscript, Sonja Brosel Performed experiments, Mercy M.Davidson Designed experiments, analysed data, Salvatore DiMauro Analysed data, revised manuscript, Eric A. Schon Analysed data, revised manuscript, Maurizio Clementi Analysed data, revised manuscript, Eva Trevisson Designed experiments, analysed data, drafted manuscript, Leonardo Salviati Designed experiments, analysed data, drafted manuscript. All authors have given final approval of the version to be published.

## Aknowldegments

This work was supported by Telethon Grants GGP06256 and GGP09207 (to L.S.), and GPP1005 (to R.R.), and by grants from Fondazione CARIPARO- Padova (to L.S. and R.R), the National Institute of Health (HD 32062) and the Marriott Mitochondrial Disorder Clinical Research Fund (to S.DM), and from NICHD (P01 HD 32062) and the Muscular Dystrophy Association (To E.A.S.).
